# Hypomethylation and Over-Expression of the Beta Isoform of BLIMP1 is Induced by Epstein-Barr Virus Infection of B Cells; Potential Implications for the Pathogenesis of EBV-Associated Lymphomas

**DOI:** 10.3390/pathogens1020083

**Published:** 2012-10-06

**Authors:** Katerina Vrzalikova, Sarah Leonard, Yichao Fan, Andrew Bell, Martina Vockerodt, Patrik Flodr, Kenneth L. Wright, Martin Rowe, Qian Tao, Paul G. Murray

**Affiliations:** 1 School of Cancer Sciences, University of Birmingham, B15 2TT, UK; E-Mails: leonarSM@adf.bham.ac.uk (S.L.); bellai@adf.bham.ac.uk (A.B.); vockerom@adf.bham.ac.uk (M.V.); rowem@adf.bham.ac.uk (M.R.); p.g.murray@bham.ac.uk (P.G.M.); 2 Laboratory of Molecular Pathology, Department of Pathology, and Institute of Molecular and Translation Medicine, Faculty of Medicine and Dentistry, Palacky University, Olomouc, 779 00, Czech Republic; E-Mail: flodrpatrik@yahoo.co.uk (P.F.); 3 The Cancer Epigenetics Laboratory, Sir YK Pao Center for Cancer, Department of Clinical Oncology, Hong Kong Cancer Institute and Li Ka Shing Institute of Health Sciences, The Chinese University of Hong Kong, 999077, Hong Kong; E-Mails: qtao@clo.cuhk.edu.hk (Q.T.); qtao@clo.cuhk.edu.hk (Y.F.); 4 H. Lee Moffitt Cancer Center, 12902 Magnolia Drive, MRC-4 East, Tampa, FL 33612, USA; E-Mail: ken.wright@moffitt.org (K.L.W.)

**Keywords:** BLIMP1, Epstein-Barr virus, hypomethylation, Hodgkin’s lymphoma

## Abstract

B-lymphocyte-induced maturation protein 1 (BLIMP1) exists as two major isoforms, α and β, which arise from alternate promoters. Inactivation of the full length BLIMP1α isoform is thought to contribute to B cell lymphomagenesis by blocking post-germinal centre (GC) B cell differentiation. In contrast, the shorter β isoform is functionally impaired and over-expressed in several haematological malignancies, including diffuse large B cell lymphomas (DLBCL). We have studied the influence on BLIMP1β expression of the Epstein-Barr virus (EBV), a human herpesvirus that is implicated in the pathogenesis of several GC-derived lymphomas, including a subset of DLBCL and Hodgkin’s lymphoma (HL). We show that BLIMP1β expression is increased following the EBV infection of normal human tonsillar GC B cells. We also show that this change in expression is accompanied by hypomethylation of the BLIMP1β-specific promoter. Furthermore, we confirmed previous reports that the BLIMP1β promoter is hypomethylated in DLBCL cell lines and show for the first time that BLIMP1β is hypomethylated in the Hodgkin/Reed-Sternberg (HRS) cells of HL. Our results provide evidence in support of a role for BLIMP1β in the pathogenesis of EBV-associated B cell lymphomas.

## 1. Introduction

The PRDM1 gene encodes two major isoforms, designated BLIMP1α and BLIMP1β, which arise from alternate promoters [1]. The full-length BLIMP1α protein orchestrates plasma cell differentiation by repressing genetic programs associated with the germinal centre (GC) stages, while at the same time activating those programs associated with plasma cell functions [2,3]. In contrast, BLIMP1β is transcribed from a promoter and exon located upstream of exon 4 of the PRDM1 gene [1]. The BLIMP1β protein lacks the first 101 amino acids of BLIMP1α and instead contains three amino acids fused to amino acids 102–789 of BLIMP1α. BLIMP1β, which lacks most of the positive regulatory (PR) domain and N-terminal acidic region, has a diminished capacity to repress target genes [1]. Since BLIMP1β contains the DNA-binding domain but bears a disrupted regulatory domain it has been suggested that it behaves as an inhibitor of BLIMP1α [1].

Other members of the PRDM family, including PRDM2 (RIZ), PRDM3 (MDS1-EVI1) and PRDM16 (MEL1) can also express a full-length protein containing the PR domain as well as a truncated protein missing the PR domain [4]. The balance of expression of these different PRDM isoforms is disrupted in many cancers and results from both the over-expression of the truncated proteins as well as the loss of expression of the full-length proteins [5,6,7,8,9,10,11,12]. For example, RIZ1, the full-length product of PRDM2, is a tumour suppressor protein because it demonstrates a loss of function in many types of human cancers with genomic deletions or point mutations and because RIZ1-deficient mice have been shown to develop diffuse large B cell lymphomas (DLBCL) [5,6,7]. In contrast, RIZ2, which lacks the PR domain, is over-expressed in breast cancer and in acute lymphoblastic leukaemias [8,9]. The transcript of the long form of the *MDS1-EVI1/PRDM3 *gene is expressed at very low levels in leukaemia cells, whereas the short form of the *EVI1 *gene is over-expressed in murine leukaemia cells with viral integration in the *EVI1 *locus and in human leukaemias with chromosome 3q abnormalities [10,11,12,13,14,15]. The human *MEL1/PRDM16 *also has two alternative protein forms, a long form, MEL1, and a short form, MEL1S. The latter is over-expressed in leukaemia cells carrying the t(1;3) translocation [16,17]. 

Inactivation of the PRDM1 gene leading to loss of BLIMP1α function occurs in a subset of DLBCL of the activated B-cell type and is believed to contribute to lymphomagenesis by blocking post-GC B cell differentiation [18,19]. In contrast, the over-expression of BLIMP1β has been reported in multiple myeloma, DLBCL and in some T cell lymphomas [1,20,21,22]. BLIMP1β over-expression is associated with advanced Ann Arbor stage and a high-risk International Prognostic Index in T cell lymphomas and with shorter patient survival in both DLBCL and T cell lymphoma patients [21,22]. In DLBCL, the increased BLIMP1β mRNA levels are associated with hypomethylation of the BLIMP1β promoter [23]. In both B- and T-cell lymphomas, BLIMP1β expression might also be associated with *in vitro* resistance to chemotherapeutic agents [21,22].

The Epstein-Barr virus (EBV) is a human herpesvirus that has been shown to be involved in the pathogenesis of several GC-derived lymphomas, including classical Hodgkin’s lymphoma (HL) and more recently a subset of DLBCL [24,25]. A previous study reported that EBV infection of myeloma cells decreased BLIMP1 expression, but this study was not able to differentiate between the different isoforms [26]. Recently we showed that EBV infection of GC B cells resulted in the down-regulation of the BLIMP1α isoform [27]. Here we have investigated the influence of EBV on the expression and methylation status of the BLIMP1β isoform. 

## 2. Results and Discussion

### 2.1. Induction of BLIMP1β Expression Following EBV Infection of Primary Human B cells

**Figure 1:**
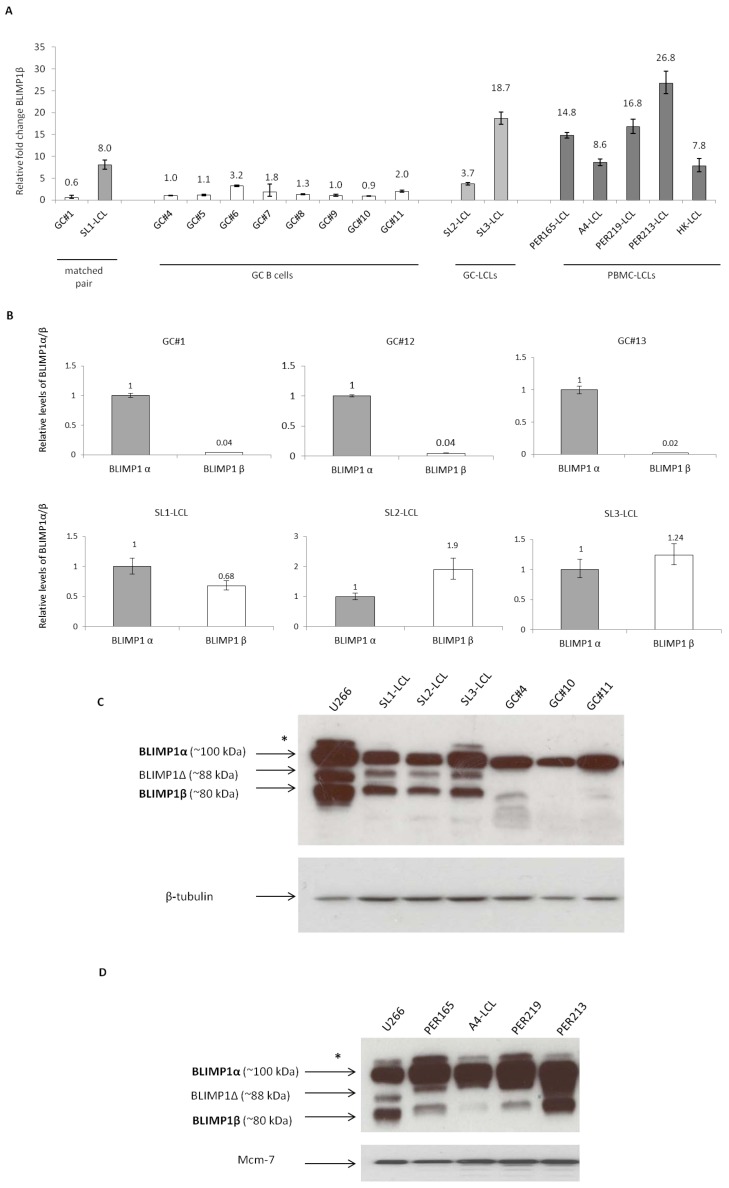
Induction of B-lymphocyte-induced maturation protein 1 (BLIMP1β) expression following Epstein-Barr virus (EBV) infection of primary human B cells.

We first explored the impact of EBV infection on BLIMP1β expression in B cells. We studied BLIMP1β expression in three LCLs derived from GC B cells as well as in five LCLs established by the PBMCs of healthy donors. The generation of the GC-derived LCLs has been described previously [28]; these LCLs were examined six weeks following infection, at which time these cells were shown to be polyclonal in nature and to express the typical Latency III pattern of EBV viral genes [28]. We found that when compared to normal un-infected GC B cells, the LCLs showed increased expression of BLIMP1β mRNA (Figure 1A). Because we had previously shown that EBV infection of B cells was accompanied by the decreased expression of the BLIMP1α isoform [27], we next compared the relative levels of each isoform in GC B cells and GC-derived LCLs. Figure 1B shows that EBV infection of GC B cells dramatically reduced the BLIMP1α:BLIMP1β ratio. For example, in the matched pair, GC#1 and SL1-LCL, the BLIMP1α:BLIMP1β ratio fell from 25 (1/0.04) in normal GC B cells to 1.47 (1/0.68) in EBV-transformed GC B cells. 

We then used an antibody that recognizes both BLIMP1α and BLIMP1β isoforms to study BLIMP1β protein expression in these samples by immunoblotting. Figure 1C shows that while normal GC B cells expressed only the BLIMP1α isoform, the GC B cell-derived LCLs expressed both BLIMP1α and BLIMP1β protein. The BLIMP1β protein consistently ran at a slightly higher molecular weight than in U266 cells which we used as a positive control. In U266 cells and in one LCL, we also observed a slightly heavier band running above the BLIMP1α isoforms (marked with *); this could represent the recently described sumoylated form of BLIMP1 [29,30]. Shorter exposure of these blots also revealed that whereas the BLIMP1α protein separated as a single sharp band in GC B cells, it appeared as a weaker double band in the GC-derived LCLs (data not shown). A further band migrating at approximately 88 kDa was also seen in the GC-derived LCLs and U266 cells, but not in GC B cells. This is likely to correspond to the recently reported BLIMP1Δ6 isoform, which migrates at this molecular weight [31,32]. We also studied BLIMP1β protein expression in four LCLs established from the PBMCs of healthy donors. Figure 1D shows that BLIMP1β was detectable in all the studied LCLs. Although BLIMP1β protein levels varied between these PBMCs-derived LCLs, they correlated well with the mRNA levels (shown in Figure 1A). The BLIMP1β protein was not detectable in normal B cells isolated from peripheral blood (data not shown).

### 2.2. The BLIMP1β-specific Promoter is Hypomethylated in EBV-infected Human Germinal Center B cells

**Figure 2:**
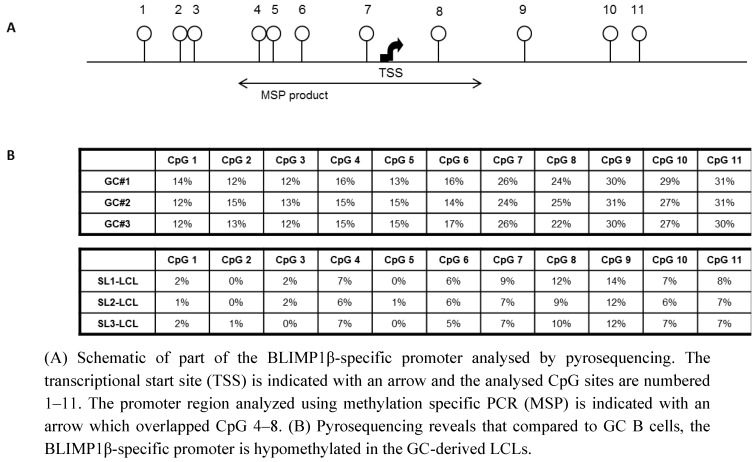
The BLIMP1β-specific promoter is hypomethylated in EBV-infected primary human germinal center B cells.

Because it has recently been shown that the increased expression of BLIMP1β observed in DLBCL is accompanied by the hypo-methylation of the BLIMP1β-specific promoter [23], we used pyrosequencing to investigate the methylation status of BLIMP1β in EBV-infected GC B cells. Figure 2 shows that relative to normal GC B cells, all 11 CpGs within the BLIMP1β-specific promoter were hypomethylated in the GC-derived LCLs. We conclude that the over-expression of BLIMP1β in EBV-infected primary human GC B cells is accompanied by the hypomethylation of the BLIMP1β-specific promoter.

### 2.3. Hypomethylation and Increased Expression of BLIMP1β in Hodgkin’s Lymphoma

**Figure 3:**
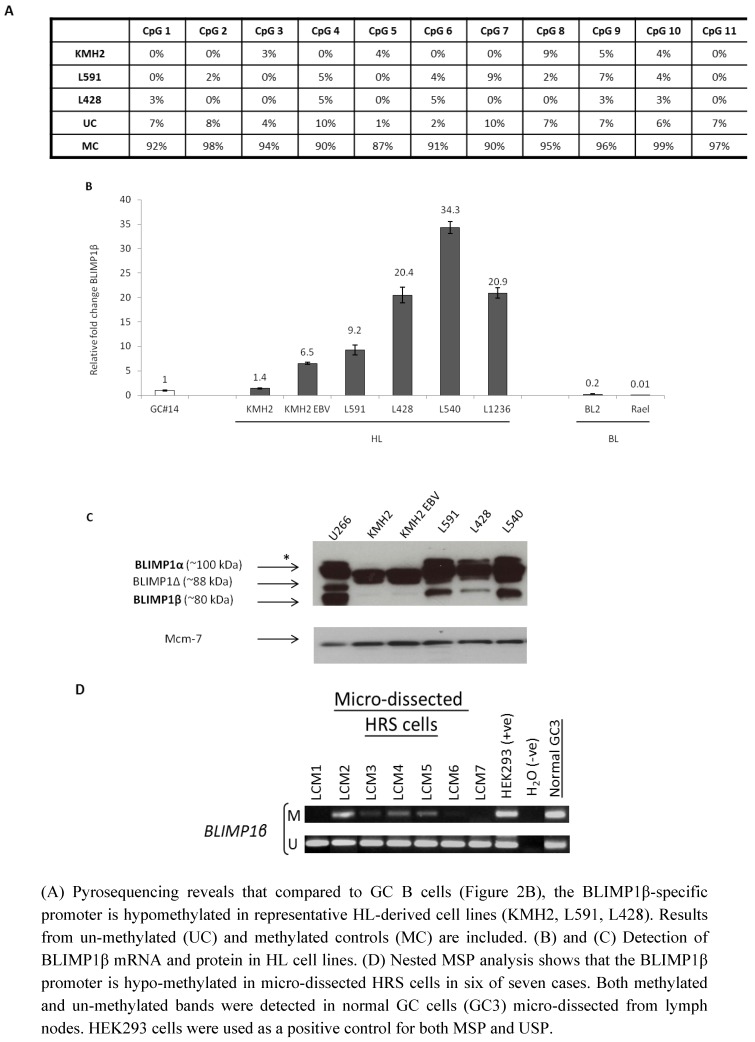
Hypomethylation of BLIMP1β in Hodgkin’s lymphoma.

**Supplementary Figure 1:**
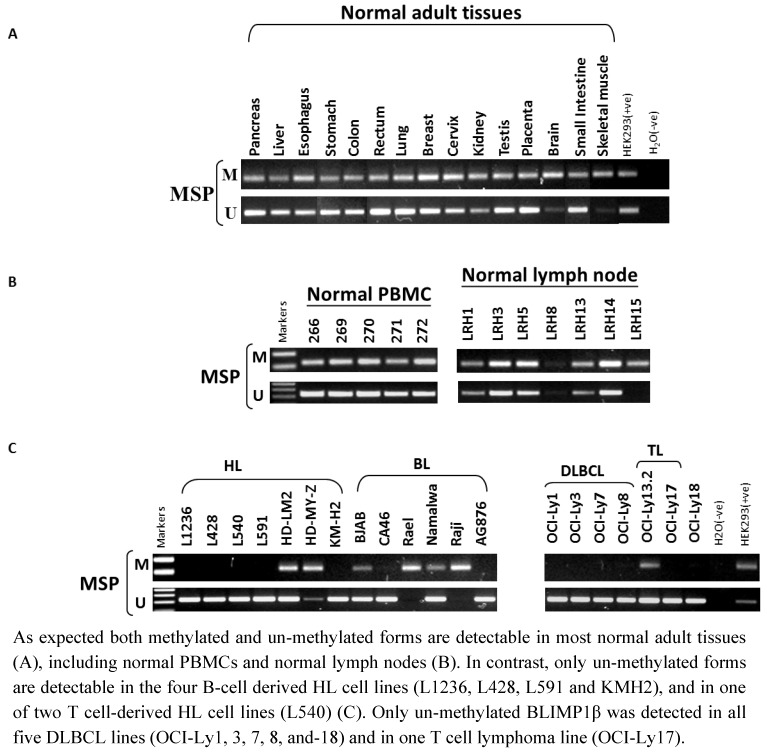
Methylation-specific PCR (MSP) analysis of the BLIMP1β-promoter in normal tissues and lymphoma cell lines.

We next used pyrosequencing to investigate the methylation status of the BLIMP1β-specific promoter in HL-derived cell lines. Figure 3A shows that the BLIMP1β-specific promoter was hypomethylated in the three HL cell lines examined (L591, L428, KMH2). We used MSP to confirm loss of methylation of the BLIMP1β-specific promoter in these three HL cell lines as well as in a further two HL cell lines (L540 and L1236) and in several DLBCL lines and T cell lymphoma-derived lines (Supplementary Figure 1). We observed that in the HL cell lines, the hypomethylation of BLIMP1β was accompanied by an increase in BLIMP1β mRNA and in most cases by an increase in BLIMP1β protein (Figure 3B, 3C). Although we found hypomethylation of BLIMP1β in KMH2 cells, these cells showed only low levels of BLIMP1β mRNA and barely detectable BLIMP1β protein (Figure 3B and 3C), suggesting that the hypomethylation of BLIMP1β is alone insufficient for BLIMP1β protein expression in this cell line. Furthermore although EBV infection of KMH2 cells increased BLIMP1β mRNA levels, there was no discernible increase in BLIMP1β protein. We also found that the Burkitt lymphoma cell lines BL2 and Rael did not express BLIMP1β mRNA (Figure 3B). Consistent with this, we found the BLIMP1β isoform to be methylated in Rael cells (Supplementary Figure 1). 

Finally, we examined the methylation status of the BLIMP1β-specific promoter in primary HRS cells which we micro-dissected from seven cases of HL. We used MSP for this analysis because conventional pyrosequencing is not sufficiently sensitive to detect methylation in small numbers of microdissected cells. Figure 3D shows that the BLIMP1β promoter was hypo-methylated in HRS cells in six of seven cases. Both methylated and un-methylated bands were detected in normal GC B cells micro-dissected from reactive lymph nodes. However, it should be noted that we observed that the BLIMP1β promoter was hypomethylated in HRS cells from both EBV-positive and EBV-negative cases of HL (Supplementary Table 3). 

### 2.4. Discussion of Results

Plasma cell differentiation is regulated by the coordinated expression of a number of transcription factors. Among these is BLIMP1, which can exist as two major isoforms, designated BLIMP1α and BLIMP1β [1]. BLIMP1α is essential for plasma cell differentiation because it extinguishes the gene-expression program of germinal center B cells, while at the same time inducing genes required for terminal differentiation [2,3]. In contrast, the truncated BLIMP1β protein has a functional DNA-binding domain but contains a disrupted regulatory domain [1]. In a previous study we reported that EBV could down-regulate BLIMP1α expression in primary human GC B cells [27]. In the present study, we show that EBV infection of primary human GC B cells is followed by the increased expression of BLIMP1β. Although we previously showed that the EBV-encoded LMP1 was sufficient to down-regulate BLIMP1α in GC B cells, we found that this viral gene does not up-regulate BLIMP1β in these cells (data not shown). 

The up-regulation of BLIMP1β in EBV-infected B cells could be of functional importance. Other truncated PRDM family members when over-expressed can act as inhibitors of their respective full-length isoform. For example, the effects of MDS1-EVI1, a PR domain containing form of the MDS1-EVI1 gene, can be reversed by over-expressing EVI1, the PR lacking isoform [11]. Furthermore, EVI1 has been shown to repress TGF-β signaling, through interaction with Smad3, while MDS1-EVI1 augments the response to the growth inhibitory effect of TGF-β [12,13,14,15]. Moreover, the truncated form of the PRDM2 gene (RIZ2), which lacks the PR domain, has been shown to inhibit trans-activation activity of the oestrogen receptor by RIZ1, the full length product of PRDM2 [33]. 

We observed that the up-regulation of BLIMP1β was accompanied by reduced expression of BLIMP1α resulting in similar levels of expression of these two isoforms in EBV infected GC-derived cells. BLIMP1β has been shown to have only 20% of the transcriptional repressive activity of BLIMP1α and can form hetero-dimers with BLIMP1α [1]. Therefore, we suppose that BLIMP1β may counteract the ability of BLIMP1α to drive plasma cell differentiation and in turn prevent induction of the EBV lytic cycle. This could be important for the subsequent development of EBV-associated lymphomas because viral replication usually leads to cell death, an event which is presumably incompatible with lymphomagenesis. 

Apart from the up-regulation of BLIMP1β, additional mechanisms might contribute to the inactivation of BLIMP1α. Our immunoblotting analysis revealed the presence of a slightly heavier protein running above the BLIMP1α isoforms in U266 cells and in several of the LCLs. This is likely to represent the recently described sumoylated form of BLIMP1α, which is rapidly degraded and could be impaired in its ability to induce plasma cell differentiation [29,30]. We also observed an intermediate band running between BLIMP1α and BLIMP1β which most likely corresponds to the BLIMP1Δ isoform created by an alternative splicing of *PRDM1 *exon 6 in humans or exon 7 in mice [31,32,34]. The resulting BLIMP1Δ protein lacks the first 3 zinc fingers and is therefore predicted to be non-functional [34]. However, despite having impaired DNA binding activity, the BLIMP1Δ form was shown to interfere with the activity of full-length BLIMP1α, presumably by forming non-functional heterodimers [31]. 

We also observed that the increased expression of BLIMP1β in EBV-transformed GC B cells was accompanied by hypomethylation of the BLIMP1β-specific promoter. These results are consistent with a previous report showing that in DLBCL, increased BLIMP1β mRNA levels are associated with BLIMP1β promoter hypomethylation [23], and also with our previous report that EBV infection of GC B cells is followed by the widespread hypomethylation of cellular genes [28]. In the latter study we showed that EBV infection of GC B cells was followed shortly afterwards by the up-regulation of the DNA methyltransferase, DNMT3A and by the down-regulation of DNMT3B and DNMT1, a pattern of expression which was recapitulated in HL [28]. It remains to be established which, if any, of the DNMTs are involved in regulating the BLIMP1β-specific promoter in EBV-transformed B cells. However, we also found that in HL cell lines, BLIMP1β promoter hypomethylation was not always accompanied by BLIMP1β protein expression suggesting that in some cases promoter hypomethylation alone may not be sufficient for protein expression. 

EBV is implicated in the pathogenesis of several GC-derived lymphomas, which include HL and a subset of DLBCL of the elderly and immunosuppressed [24,25]. In the present study, we not only confirmed previous reports that the BLIMP1β promoter is hypomethylated in DLBCL cell lines [23] but we also showed that BLIMP1β is frequently hypomethylated in the HRS cells of HL. Immunohistochemistry has been used previously to show that BLIMP1 protein is expressed in HRS cells in some cases of HL [35,36,37,38]. Although, it would be of interest to know which BLIMP1 isoform is expressed in primary HRS cells, an antibody, which can differentiate between the different BLIMP1 isoforms is not available at the present time.

We observed BLIMP1β promoter hypomethylation in HRS cells from both EBV-positive and EBV-negative cases of HL. These data suggest that the over-expression of BLIMP1β could be involved in the pathogenesis of both EBV-positive and EBV-negative tumours; BLIMP1β might be required for the arrest of terminal differentiation which is generally regarded as an important component in the pathogenesis of most, if not all, GC-derived B cell lymphomas. However, an alternative possibility is that the induction of BLIMP1β expression is a tumor- or cell cycle-related phenomenon and occurs independently of EBV infection in HRS cells. Further studies are required to determine if and how BLIMP1β contributes to B cell lymphomagenesis and also to identify the mechanisms responsible for the regulation of BLIMP1β hypomethylation and expression in Hodgkin’s lymphoma.

## 3. Experimental Section

### 3.1. Cells

The GC B cells were obtained from patients undergoing routine tonsillectomy. The peripheral blood mononuclear cells were obtained from healthy volunteers. LCLs were established by infecting tonsillar GC B cells isolated from three separate donors with 2089 wild type EBV [28] and are referred to throughout as SL1-LCL, SL2-LCL, and SL3-LCL. LCLs derived from peripheral blood mononuclear B cells (PBMCs) PER213-LCL, PER219-LCL, PER165-LCL, A4-LCL, HK-LCL were established using the reference EBV strain B95.8 and were a gift of Dr Heather Long and Dr Nikki Smith (University of Birmingham, United Kingdom). KMH2, L428, HDLM2, HD-My-Z, L1236, L540 are EBV-negative HL cell lines [39,40,41,42,43,44]. L591 is EBV-positive HL cell line [45]. KMH2 EBV is derived from EBV-negative KMH2 cells infected with Akata-derived recombinant virus and maintained under geneticin selection [24]. BL2, CA46 and BJAB are EBV-negative BL cell lines [46,47,48]. Rael, Raji, Ag876 and Namalwa are EBV-positive BL cell lines [49,50,51,52]. OCI-Ly1, 3, 7, 8, and-18 are EBV-negative DLBCL lines [53]. OCI-Ly17 and Oci-Ly13.2 are EBV-negative T cell lymphoma lines [53]. U266 are multiple myeloma cells which served as a positive control for BLIMP1 expression [54]. All cell lines were cultured at 37 °C in 5% CO_2_ in RPMI1640 growth media supplemented with 10% foetal calf serum, 2mM L-glutamine and 1% penicillin-streptomycin solution. 

### 3.2. Reverse-Transcriptase-PCR

RNA was extracted using RNeasy Mini Kit or Micro Kit including removal of genomic DNA with RNase-Free DNase Set (QIAGEN). cDNA was generated in a reaction consisting of 400 ng of RNA, 250 ng of random primers (Promega), 10 mM dNTP Mix (Roche Diagnostics) and SuperScript® III Reverse Transcriptase (Invitrogen) following the protocol supplied by the manufacturer. If required, cDNA was purified using GenEluteTM PCR Clean-Up kit (Sigma-Aldrich).

### 3.3. Quantitative PCR

All real-time PCR assays were performed using an ABI Prism 7700 sequence detection system (Applied Biosystems). A final reaction volume of 25 µL contained 1 × TaqMan universal PCR mastermix (Applied Biosystems), 2.5–25.0 pmol primers, 5 pmol probe, 1.5 µL of house-keeping assay and 5 µL cDNA (equivalent to required ng input of RNA). Thermal-cycling conditions were: 2 minutes at 50 °C, 12 minutes at 95 °C and 40–50 rounds of 15 seconds at 95 °C and 1 minute at 60 °C. All test samples were run in triplicate and template-negative reactions served as controls. The probe targeting BLIMP1β isoform was published elsewhere [20], but for our study it was re-labelled with minor groove binder (MGB™) reporter dye at the 5’ end and non-fluorescent quencher (NFQ) at the 3’ end and purchased from Applied Biosystems. All other real-time PCRs were performed using commercially available assays (Supplementary Table 1). The 2-Delta-Delta CT method was used to quantify expression relative to the housekeeping control. The normalized values were expressed relative to the reference sample, which was set to a relative quantity value of 1 [55].

### 3.4. Bisulphite Modification and Pyrosequencing

Genomic DNA (500 ng) was bisulphite converted using the EZ DNA methylation kit (Zymo Research). All pyrosequencing primers were designed using Biotage PSQ primer design software. Biotinylated, non-biotinylated and sequencing primers are listed in Supplementary Table 2. The PCR was performed in a total volume of 50 µL using 25 µL hotstart taq master mix (Thermo Scientific), 5 pmol biotinylated primer, 10 pmol non-biotinylated primer and 10 µL bisulphite modified DNA. The pyrosequencing reactions were performed on a Pyromark ID system (Biotage) and analysed using Pyro Q-CpG software (Biotage). 100ng unmethylated control (UC) and methylated control (MC) DNA (Millipore) was bisulphite modified and run with each pyrosequencing reaction.

### 3.5. Methylation-Specific PCR (MSP)

Genomic DNA was treated with sodium meta-bisulphite (Sigma) as previously described [56], but without restriction endonuclease digestion. MSP was performed according to our previous method [57]. Methylation-specific primers were: for methylated promoter (221-bp product), PRDM1bm1 (5’-ATTTAGTTTGACGTCGTTAGTC-3’) and PRDM1bm3 (5’-TTATCGTCTTTTCATATTCG-3’); for unmethylated promoter (227-bp product), PRDM1bu1 (5’-GATTTAGTTTGATGTTGTTAGTT-3’) and PRDM1bu3 (5’-CAATTTTATCATCTTTTCATATTCA-3’). The MSP primers amplified a product overlapping CpG 4–8 as indicated on Figure 2. They did not amplify any DNA without bisulphite treatment and therefore were shown to be specific. For each sample, 0.5 µL of bisulphite-treated DNA (~25 ng measured before bisulphite treatment) was PCR amplified using 0.3125 U of AmpliTaq Gold (Perkin Elmer, Norwalk, CT) for cell line DNA (or 0.46875 U of AmpliTaq Gold for normal PBMCs, lymph node, and primary HL DNA), with 2 mmol/L MgCl_2_, 0.2 mmol/L dNTP and 0.6 umol/L each primer in a 12.5 µL reaction volume. MSP was conducted with hotstart taq master mix at 95 °C for 10 min, then 41 cycles (94 °C, 30s; 58 °C, 30s; 72 °C, 30s) for MSP, or 40 cycles (94 °C, 30s; 58 °C, 30s; 7 °C, 30s) for USP, followed by 72 °C for 5 mins. MSP products were analyzed on 2% agarose gel. A normally methylated gene, *ANKRD30A*, was also used as a positive control for those samples in which BLIMP1β methylation was undetectable. 

### 3.6. Methylation Analysis of Micro-dissected HRS Cells by Nested-MSP

Two hundred HRS cells were micro-dissected from CD30-stained cryosections of each HL case using the PALM Microbeam (Carl Zeiss MicroImaging GmbH). Only CD30 positive cells with HRS morphology were microdissected. DNA was extracted from these cells using the QIAamp DNA Mini kit. DNA was then bisulphite-treated and amplified. For nested-MSP detecting methylated alleles, first-round PCR was performed using methylation-specific primers PRDM1bm1 and PRDM1bm3 (95 °C for 10 min, 40 cycles (94 °C, 30 s; 58 °C, 30 s; 72 °C, 30 s), followed by 72 °C for 5 mins), using 0.3125 U of AmpliTaq Gold in a 6.25 ml reaction. 3 ml of 10x diluted PCR products were used for a nested-MSP detecting methylated product (113-bp) with PRDM1bm1 and PRDM1bm2 (5’-TACTACAATAAATAACAAATAAACG-3’), 0.625 U of AmpliTaq Gold in a 12.5 µL reaction. PCR was performed at 95 °C for 10 min, 65 cycles (94 °C, 30 s; 53 °C, 30 s; 72 °C, 30 s), followed by 72 °C for 5 mins. For nested-MSP detecting unmethylated alleles, primers PRDM1bu1 and PRDM1bu3 were used for the first-round PCR (40 cycles, with 0.3125 U of AmpliTaq Gold in a 6.25 µL reaction). 3 mL of 10× diluted PCR products was used in nested-MSP (114-bp product) with unmethylation-specific primers PRDM1bu1 and PRDM1bu2 (5’-TACTACAATAAATAACAAA TAAACA-3’) (65 cycles, with 0.625 U of AmpliTaq Gold in a 12.5 µL reaction). 

### 3.7. Immunoblotting

Cells lysates were prepared in RIPA buffer (10 mM Tris-HCL (pH 8.0); 140 mM NaCl; 1 mM EDTA; 1% triton, 0.1% SDS, 0.1% Sodium deoxycholate, protease inhibitors) and briefly sonicated. Protein was quantified using Bio-Rad DC Protein Assay Kit (Bio-Rad). 30 ug of U266 lysate or 75 ug of LCLs and GC B cells lysates were combined with 2× Laemmli sample buffer before SDS-PAGE (8% gel) and transferred to 0.45 m nitrocellulose transfer membrane (Protan BA85 membrane). The membrane was incubated with BLIMP1 antibody (Cell Signalling, 9115, rabbit polyclonal) diluted in 5% non-fat milk powder in TBS-Tween-20 (1:1,000) at 4 °C overnight. After a PBS-Tween-20 (0.1%) wash, blots were incubated for 30 minutes with anti-rabbit HRP-conjugated secondary IgG (Dako, 1:2,000). Anti-beta Tubulin (HRP-conjugated) antibody (Abcam, ab21058, rabbit polyclonal) or anti-Mcm-7 (Sigma-Aldrich, M7931, mouse monoclonal), both diluted in 5% non-fat milk powder in TBS-Tween-20 (1:2,000) were applied at room temperature for 1 hour. After a PBS-Tween-20 (0.1%) wash, detection was performed with enhanced chemiluminescence (GE Healthcare).

## 4. Conclusions

EBV infection leads not only to the down-regulation of BLIMP1α but also to the up-regulation of BLIMP1β. The up-regulation of BLIMP1β is associated with hypomethylation of the BLIMP1β specific promoter. Figure 1 Table 1 Table 2 Table 3

## Appendix

**Supplementary Table 1:** Commercial Real-time-PCR assays.

Gene name	Applied Biosystem Gene expression assay
BLIMP1 (PRDM1) α isoform	Hs01068508_m1
GAPDH	4310886E
β2m	4310884E

**Supplementary Table 2:** Primers used in pyrosequencing.

primer	sequence
PCR forward biotinylated primer (region 1)	TAGGTTTGGTTAGTGA
PCR reverse non biotinylated primer (region 1)	CACTTTTATCTTTCCA
Sequencing primer (region 1)	TTTTATCAATTTTTCC
PCR forward non biotinylated primer (region 2)	GGTGGAGGATAGTTGA
PCR reverse biotinylated primer (region 2)	AAATAAACCAAATTCC
Sequencing primer (region 2)	TGTATAGTTGTTTGGG

**Supplementary Table 3:** EBV status of primary HL cases micro-dissected for analysis of BLIMP1β methylation in HRS cells.

LCM case No.	Original MD HRS case No.	EBV status	BLIMP1βmethylation
LCM1	4/054	−	U
LCM2	4/133	−	M
LCM3	05/452	−	W
LCM4	05/363	+	W
LCM5	05/009	−	W
LCM6	05/092	+	U
LCM7	05/494	−	U

Key: U = unmethylated; M = methylated; W = weakly methylated.
